# Life stages and morphological variations of *Limnocythere inopinata* (Crustacea, Ostracoda) from Lake Jiang-Co (northern Tibet): a bioculture experiment

**DOI:** 10.3897/zookeys.1011.56065

**Published:** 2021-01-18

**Authors:** Can Wang, Hailei Wang, Xingxing Kuang, Ganlin Guo

**Affiliations:** 1 Guangdong Provincial Key Laboratory of Soil and Groundwater Pollution Control, School of Environmental Science and Engineering, Southern University of Science and Technology, 518055, Shenzhen, China; 2 MNR Key Laboratory of Saline Lake Resources and Environment, Institute of Mineral Resources, Chinese Academy of Geological Sciences, 100037, Beijing, China; 3 Shenzhen Municipal Engineering Lab of Environmental IoT Technologies, Southern University of Science and Technology, 518055, Shenzhen, China; 4 Jiangsu Key Laboratory of Marine Biotechnology, Jiangsu Ocean University, 222005, Lianyungang, China

**Keywords:** Cytheroidea, growth pattern, morphological characteristics, Tibetan Plateau

## Abstract

*Limnocythere inopinata* (Baird, 1843) is a Holarctic species, abundant in a number of Recent and fossil ostracod assemblages, and has many important taxonomic and (paleo)ecological applications. However, the life cycle and morphological characteristics of the living *L. inopinata* are still unclear. A bioculture experiment was designed to study life stages and morphological variations from stage A-8 to adult in this species. The living animals were collected from Lake Jiang-Co, in the northern Tibetan Plateau. Results reveal that this species possesses a special growth pattern with the maximum size increase occurring at the transition from the instars A-5 to A-4. The growth pattern deviates from Brooks’ rule and one population from Lake Dali, eastern Mongolian Plateau. This suggests that the life history of *L. inopinata* may be influenced by environmental factors. Some morphological differences between Lake Jiang-Co and European populations of *L. inopinata* are also uncovered. Therefore, a detailed morphological description of this population is provided, but refrain from erecting a new species at the present stage because those differences appear to be inconsistent.

## Introduction

*Limnocythere inopinata* (Baird, 1843), belonging to the family Limnocytheridae, is widely distributed in the Holarctic. Living populations of *L. inopinata* have been reported from Europe, Asia, and Africa. The European populations include: Austria (Löffler 1990; Yin et al. 1999; Rossi et al. 2010), Germany (von Grafenstein et al. 1999; Frenzel et al. 2010; Scharf et al. 2013), Greece (Frogley et al. 2001), Iceland (Alkalaj et al. 2019), Italy (Rossi et al. 2010; Marta et al. 2017), Poland (Szlauer-Łukaszewska 2014, 2015a, b), Spain (Mezquita et al. 1999; Rodríguez-Pérez and Baltanás 2008; Martínez-García et al. 2015), Sweden (Iglikowska and Namiotko 2012), Switzerland (Decrouy et al. 2012, 2014), Turkey (Külköylüoğlu et al. 2016; McCormack et al. 2019), and United Kingdom (Benzie 1989; Holmes et al. 2010). The Asian populations were recorded from China (Mischke et al. 2003; Zhang et al. 2015; Akita et al. 2016), India (Kramer et al. 2014), Israel (Mischke et al. 2012), and Jordan (Mischke et al. 2012). Finally, the African populations are known from Algeria (Ghaouaci et al. 2017), Egypt (Keatings et al. 2010), and Nigeria (Roberts et al. 2002).

The ecological characteristics of *L. inopinata* have been commonly used to infer paleoecological conditions (e.g., Shen et al. 2001; Poberezhnaya et al. 2006; McCormack et al. 2019). This species has been reported from a variety of water bodies (Geiger 1994), including ponds, swamps, lakes, and rivers. It is commonly distributed in shallow waters, while a few, less abundant, populations were found in deep water, e.g., from a depth of 64 m in the Baltic Sea (Hiller 1972). *Limnocythere inopinata* has salinity preference range between 0.50‰ and 9.00‰ (Usskilat 1975; Kempf 1986; Holmes et al. 1999) and a wide temperature range between 0.50 °C and 35.00 °C (Karan-Žnidaršič and Petrov 2007; Frenzel et al. 2010; Külköylüoğlu et al. 2016). *Limnocythere inopinata* from the Tibetan Plateau (TP) is especially noted for its preference of polyhaline waters (> 10‰; see Akita et al. 2016). The carapace morphology of this species is influenced by environmental factors (Carbonel et al. 1988; Yin et al. 1999). Yang et al. (2008) reported that extremely high silica values in low salinity water bodies may cause knot(s) in the shell of *L. inopinata*. Despite these studies, the life cycle and morphological characteristics of this species are still unknown.

The ontogeny of the Ostracoda is important for understanding their evolution. Watabe and Kaesler (2004) noted that the degree of adherence to Brooks’ rule during the ontogeny of the Ostracoda is related to the heterochrony in evolution. Brooks’ rule was the first estimation of growth patterns in crustaceans, and it predicts that animals will double in volume following each moult and thus present the linear dimension of growing by the cube root of two, i.e., 1.26 (Brooks 1886; Teissier 1960).

Here we study a biocultured population of *L. inopinata* collected from Lake Jiang-Co. The aim was to clarify the life stages and morphological variations from A-8 stage to adult, and to compare our data on growth rate (length and height measurements of the shell) with the Brooks’s rule and with the growth rate of the same species from Lake Dali, eastern Mongolian Plateau (MP) (Zhai et al. 2015). We also describe shell and soft parts of the Lake Jiang-Co population and compare this with the European population (Yin et al. 1999; Meisch 2000).

## Materials and methods

**Figure 1.**
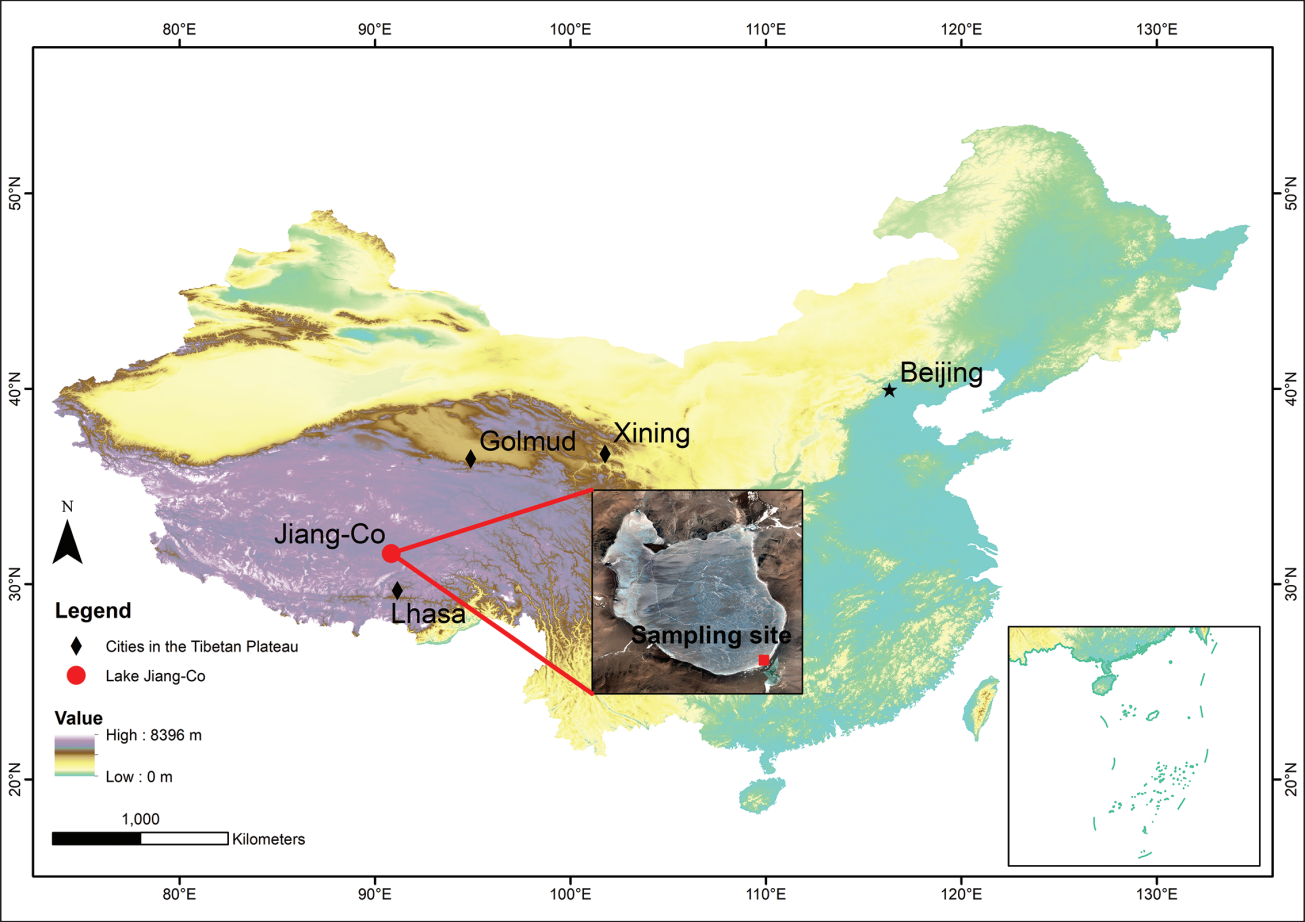
Map of the location of Lake Jiang-Co and sampling site.

Lake Jiang-Co (31°31'–31°35'N, 90°47'–90°52'E) is a brackish water lake in northern TP (Fig. 1). The lake area is approximately 40.29 km^2^. There are no inflow and outflow rivers. In this region, annual mean precipitation ranges between 300 and 600 mm, annual mean evaporation is greater than 1000 mm, and the annual mean temperature ranges between -0.30 and 2.40 °C from 1987 to 2016 according to Yang et al. (2018).

Living *L. inopinata* were collected with surface sediments from the shores of Lake Jiang-Co in September 2016, using a plankton net with a mesh size of 200 μm. Salinity was 0.59‰ and the temperature of the bottom water was 17.40 °C. Specimens, all females, were sorted under the Nikon 90i. No males were recorded in the samples. Individuals were cultured from indoor temperatures (from 9 to 14 °C) in the laboratory. The culturing water was collected from the lake and filtered through 10–20 µm filter papers. Culture was fed with *Chlorella vulgaris*.

*Limnocythere inopinata* individuals were hand-picked from biocultured populations, covering all eight juvenile stages (A-8 to A-1) and the adult stage (A). The A-8 to A stages were preliminarily determined by different size range of the left valve (i.e., length and height), based on approximately 200 individuals. Carapace length and height of every stage were measured under the microscope Nikon SME 1500 equipped with NIS-Element BR 3.1. In order to ensure that we found the true A-1 and A stages, twenty A-1 individuals and twenty adults were separately placed in eight glass dishes to observe if these individuals moulted and grew. If most of A-1 individuals moulted, and most of adults did not moult, it suggested that our division of nine stages could be trusted. We observed that most of A-1 individuals moulted and most of the adults did not do so. The size range of the left valve of A-1 and A stages was finally confirmed by the mean value of moulted A-1 individuals and non-moulted adults. Microscope and Scanning Electron Microscope (SEM) photographs of A-8 to A stages were taken by Nikon SME 1500 and Zeiss Ultra Plus SEM, respectively.

The estimation method of growth ratios of mean length and height for each ontogenetic transition follows Forel (2015):

*K_L_* = *L_m_*_+1_ / *L_m_* ,

*K_H_* = *H_m_*_+1_ / *H_m_* ,

where *K_L_* and *K_H_* are growth ratios of mean length and height, *L_m_* and *H_m_* are respectively mean length and height at stage *m*, and *L_m_*_+1_ and *H_m_*_+1_ are respectively mean length and height at stage *m*+1. We also conducted a linear regression and Pearson’s chi-squared test on all data of carapace sizes.

The adults of *L. inopinata* were dissected under 96% ethanol in the glass dish and the soft parts were sealed by 96% ethanol in the glass bottle according to Namiotko et al. (2011), and their appendages were photographed. The descriptive terminology for the hard and soft parts followed Meisch (2000). All specimens are deposited in the Key Laboratory of Saline Lake Resources and Environment, Institute of Mineral Resources, Chinese Academy of Geological Sciences, Beijing, China.

## Results

### Life stages of *L. inopinata*

**Figure 2.**
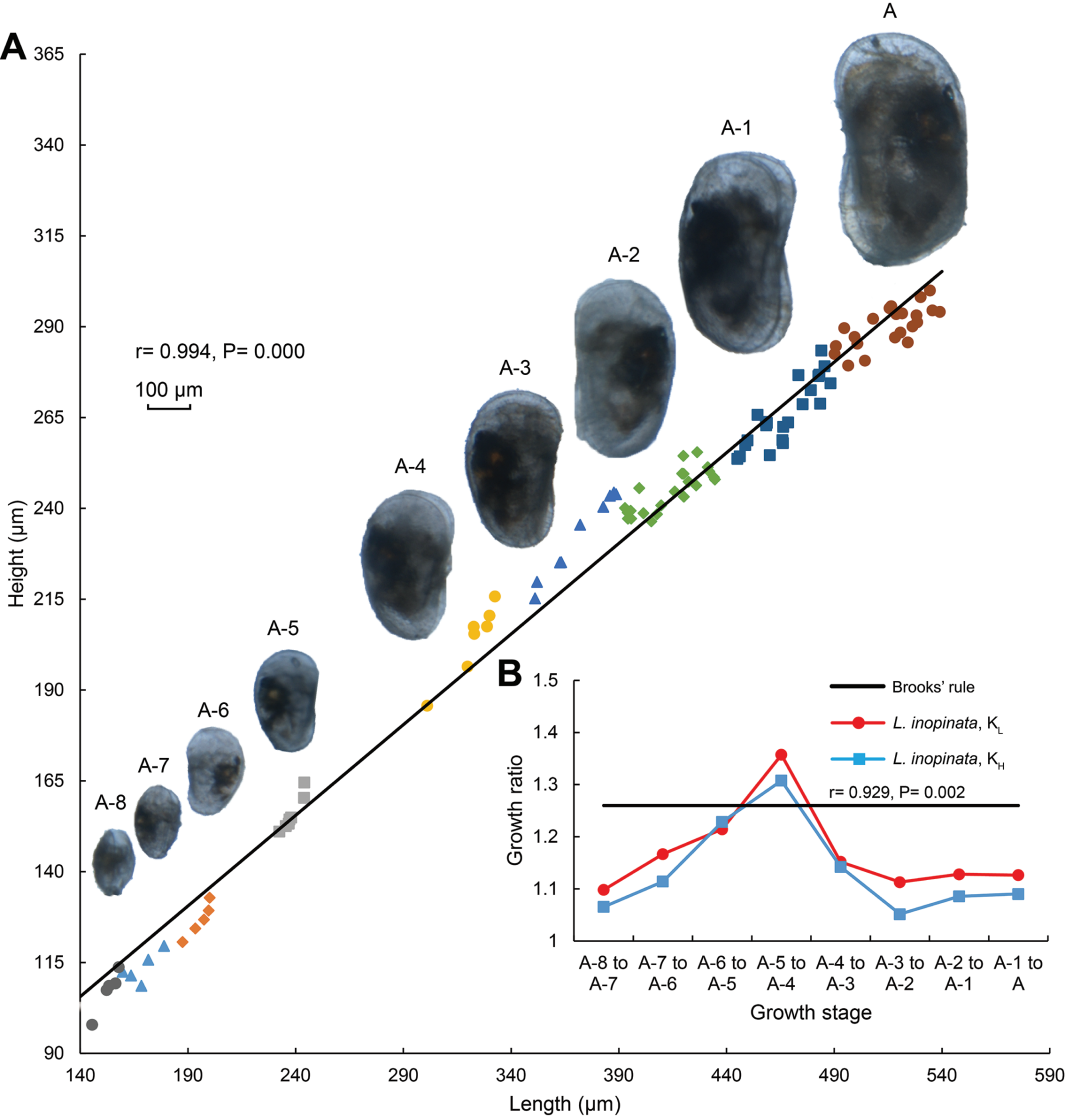
**A** Length versus height of left valves and microscope photographs of *L. inopinata* from A-8 stage to adult **B** Plot demonstrating the growth ratio elements *K_L_* and *K_H_* of *L. inopinata* from A-8 stage to adult. *K_L_* = *L_m_*_+1_ / *L_m_*, *K_H_* = *H_m_*_+1_ / *H_m_*, where *K_L_* and *K_H_* are growth ratios of mean length and height, *L_m_* and *H_m_* are respectively mean length and height at stage *m*, and *L_m_*+1 and *H_m_*+1 are respectively mean length and height at stage *m*+1. The black line suggests a growth ratio of 1.26, the value predicted by Brooks’ rule. All individuals are from the indoor bioculture laboratory.

Length versus height of left valves and microscope photographs of *L. inopinata* covering groupings of instars A-8 to A-1 and adults are shown on the Fig. 2A. Growth ratios of mean length and height of this species from A-8 stage to adult compared with Brooks’ rule are presented on the Fig. 2B.

As the individuals grew, carapace length increased, ranging from 0.153 mm to 0.526 mm. Shell height also increased, ranging from 0.107 mm to 0.209 mm (Table 1). Individual sizes were variable within a certain growth stage: that of the instar A-4 to the adult stage were more variable than those of the A-8 to the A-5. Instar A-5 clearly separated from the instars A-4 and A-6, while there were some small overlaps occurring in contiguous growth stages, such as between the instars A-2 and A-3. Carapace length displayed a significant positive correlation with the height (r = 0.994, P = 0.000).

**Table 1.** Mean length and height of left valves of *L. inopinata* from A-8 stage to adult.

Stage of growth	n	Size	
		Length (mm)	Height (mm)
adult	12	0.526 ± 0.025	0.290 ± 0.009
A-1	12	0.467 ± 0.022	0.266 ± 0.012
A-2	12	0.414 ± 0.021	0.245 ± 0.008
A-3	9	0.372 ± 0.021	0.233 ± 0.017
A-4	7	0.323 ± 0.021	0.204 ± 0.018
A-5	7	0.238 ± 0.006	0.156 ± 0.005
A-6	5	0.196 ± 0.008	0.127 ± 0.006
A-7	5	0.168 ± 0.009	0.114 ± 0.005
A-8	5	0.153 ± 0.008	0.107 ± 0.009

The *K_L_* and *K_H_* values for *L. inopinata* during ontogeny were generally lower than predicted by Brooks’ rule except for the instars A-5 to A-4. Values ranged from 1.098 to 1.357 for *K_L_*, and from 1.052 to 1.308 for *K_H_* (Table 2), and were significantly positively correlated (r = 0.929, P = 0.002). Gradually increase was observed in *K_L_* and *K_H_* during the instars A-8 to A-4, followed by small change from the instar A-4 to the adult stage. The maximum values of *K_L_* and *K_H_* occurred at the transition from the instars A-5 to A-4, and were higher than 1.26, as predicted by Brooks’ rule. The growth pattern of *L. inopinata* does not appear to follow the predictions using Brooks’ rule.

**Table 2.** *K_L_* and *K_H_* of *L. inopinata* from A-8 stage to adult.

Phase of growth	Growth ratio	
	K_L_	K_H_
A-1 to adult	1.126	1.090
A-2 to A-1	1.128	1.086
A-3 to A-2	1.113	1.052
A-4 to A-3	1.152	1.142
A-5 to A-4	1.357	1.308
A-6 to A-5	1.214	1.228
A-7 to A-6	1.167	1.114
A-8 to A-7	1.098	1.065
Mean growth ratio	1.169	1.136

**Figure 3.**
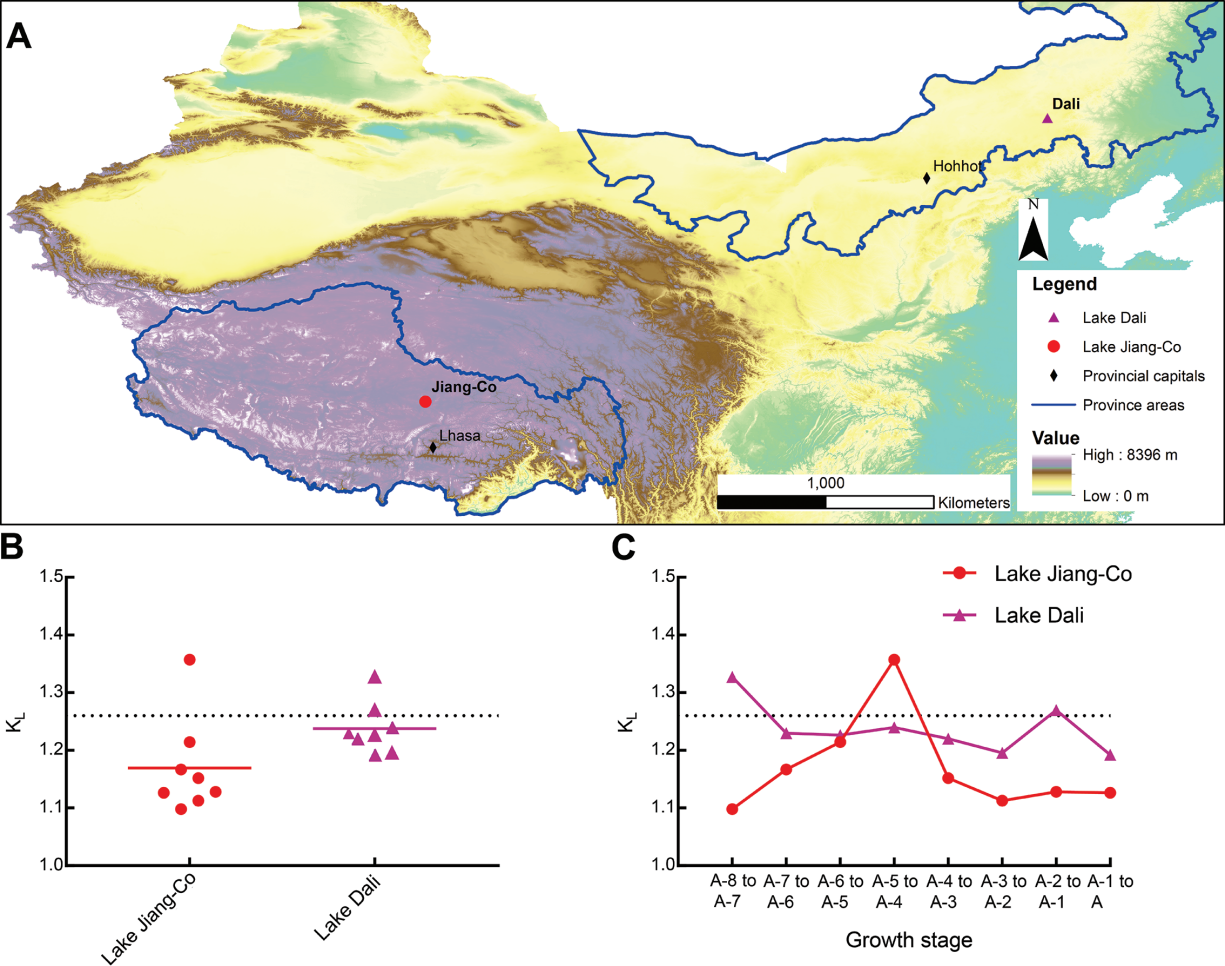
**A** Locations of Lake Jiang-Co and Lake Dali **B** scatter plot with mean value of *K_L_* of *L. inopinata* populations from A-8 stage to adult in Lake Jiang-Co and Lake Dali (Zhai et al. 2015). The rounded dot and triangle represent the growth ratio between adjacent instars **C** comparison between *K_L_* of *L. inopinata* populations from A-8 stage to adult in Lake Jiang-Co and Lake Dali (Zhai et al. 2015). The grey dotted lines suggest a growth ratio of 1.26, the value predicted by Brooks’ rule.

The *K_L_* values of the *L. inopinata* population in Lake Jiang-Co from the A-8 to the adult was compared with values obtained for a population from Lake Dali (Zhai et al. 2015) (Fig. 3), a marginal lake, located at the eastern part of MP (43°13'–43°23'N, 116°29'–116°45'E) with a surface area of 192 km^2^ (Zhai et al. 2015) (Fig. 3A). Generally, the mean *K_L_* values for both populations were lower than predicted by Brooks’ rule, but that for the population from Lake Dali were higher than that from the Lake Jiang-Co (Fig. 3B). In addition, the growth pattern observed in Lake Dali was less varied: *K_L_* varies from 1.153 to 1.327, and the maximum value of *K_L_* was observed at the transition from the instar A-8 to the instar A-7. The maximum *K_L_* was followed by a remarkable decline to a low *K_L_*, followed by a relatively stable value, demonstrating that the *L. inopinata* populations in Lake Jiang-Co and Dali experience different growth patterns.

#### Morphological description of *Limnocythere inopinata*

Figures 4, 5

**Material examined and locality.** Shells of *L. inopinata* from the instar A-8 to the adult stage collected from an indoor biocultured population, which was originally sampled from Lake Jiang-Co. The appendages of six adult females were selected for examination.

**Figure 4.**
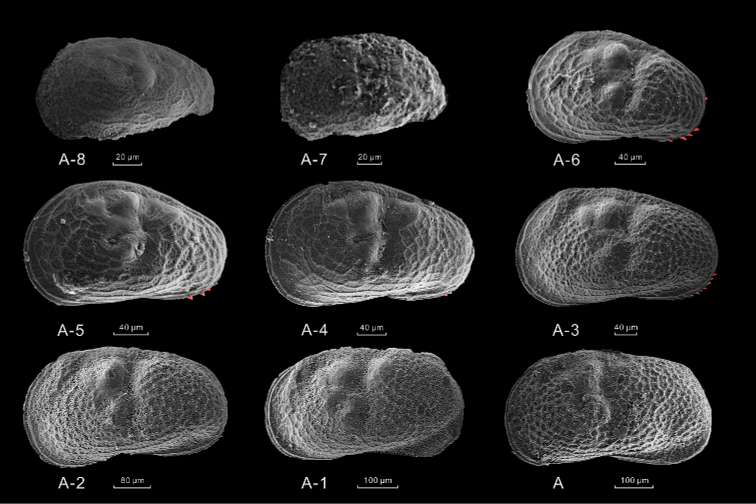
SEM photographs of *L. inopinata* describing carapace morphological features of different developmental stage. All the left valves are from indoor bioculture. The red parts of the instars A-6 to A-3 represent the marginal denticles.

**Description of shell.** All the valves were thin and semi-transparent (Fig. 4). Generally, the carapace of instars A-8 and A-7 were too soft to remain intact after dissection. These individuals were marginally broken in ventral view, but the valve surface was ornamented with rounded pits and a reticulate pattern of ridges. Each valve had a medio-dorsal transverse groove at approximately the middle of its length. The groove of the instars A-6 to the adult stage were more obvious than that of the instars A-8 and A-7. The dorsal margin was slightly rounded in the instars A-8 to A-3 and almost straight in the instar A-2 to the adult stage. The valves of the instars A-6 to A-3 had a clear postero-ventral row of marginal denticles, while that trait was faint in the A-2 instar to the adult stage.

**Figure 5.**
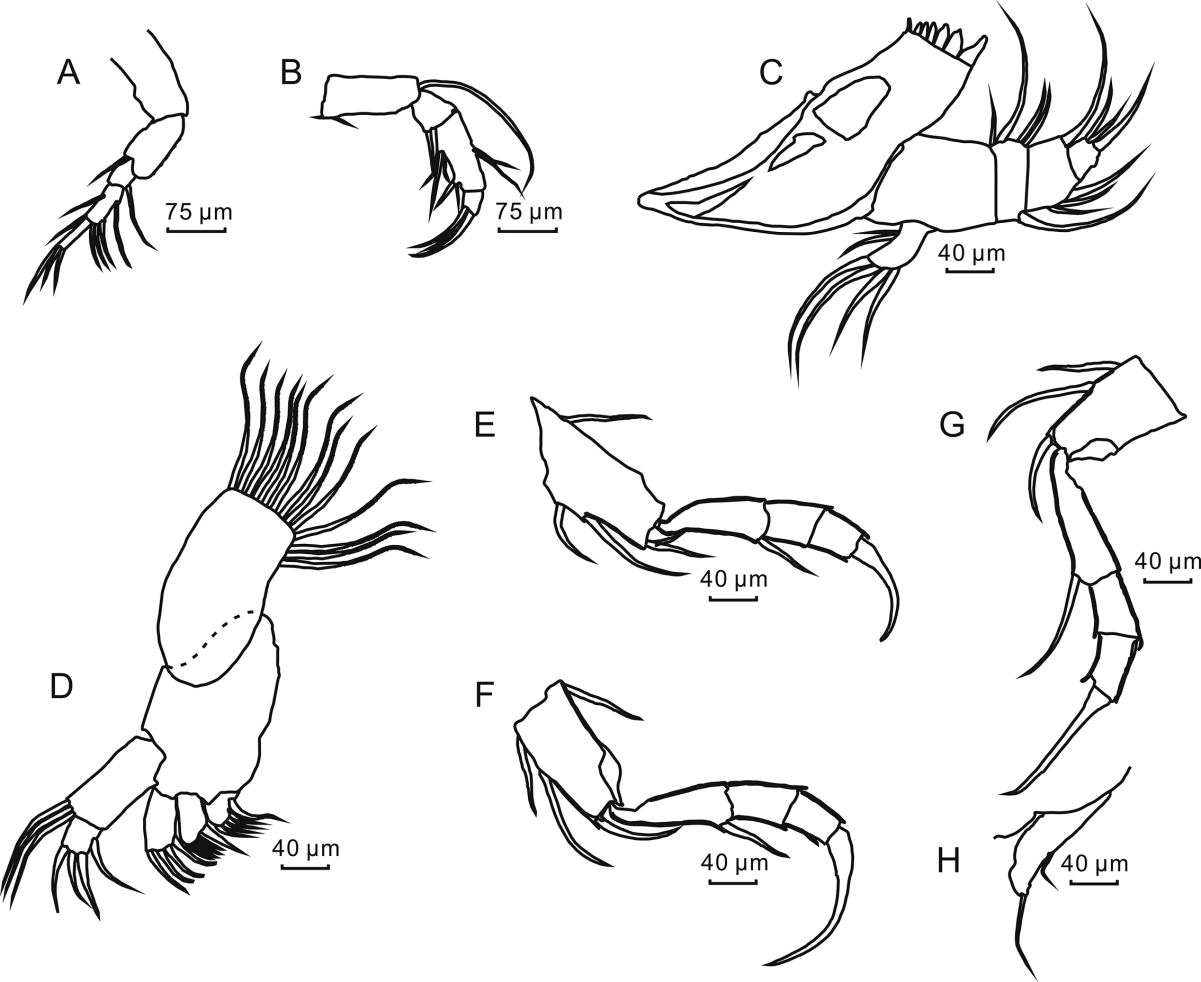
Appendage morphology of *L. inopinata*, adult female **A** antennule **B** antenna **C** mandible **D** maxilla **E** first thoracopod **F** second thoracopod **G** third thoracopod **H** uropodal rami.

**Description of the soft parts of adult. *Antennule*** (Fig. 5A) symmetrical, uniramous, 5-segmented. The lengths of the segments, from proximal to distal ones, being 73.6 μm, 58.2 μm, 18.0 μm, 39.8 μm, and 33.4 μm, respectively (Table 3). Armature of segments as follows: segment II with one antero-apical seta; segment III with one postero-apical seta; segment IV with one postero-medial seta, and one anterior and three posterior setae at the apical part; segment V with three claws, lengths 53.9 μm, 41.4 μm, and 41.2 μm, respectively.

**Table 3.** Length of adult appendage segments and terminal segment claws of *L. inopinata*. A1, antennule; A2, antenna; T1, first thoracopod; T2, second thoracopod; T3, third thoracopod; UR, uropodal rami.

Appendage	Segment I (μm)	Segment II (μm)	Segment III (μm)	Segment IV (μm)	Segment V (μm)	Claw 1 (μm)	Claw 2 (μm)	Claw 3 (μm)
A1	73.6	58.2	18. 0	39.8	33.4	53.9	41.4	41.2
A2	78.6	34.5	66.1	18.0	–	67.9	57.5	52.1
T1	64.3	51.6	24.7	27.9	–	81.4	40.4	–
T2	58.0	54.7	20.4	29.6	–	67.7	–	–
T3	62.0	57.0	25.2	25.7	–	95.4	–	–
UR	39.2	–	–	–	–	–	–	–

***Antenna*** (Fig. 5B) biramous, with a nearly rectangular protopod, 78.6 μm in length (Table 3) with two ventro-basal setae. The exopod degenerated to a spinneret seta. The endopod well-developed and consisting of three segments, 34.5 μm, 66.1 μm, and 18.0 μm in length. Armature of endopod segments as follows: segment I with one postero-apical seta; segment II with two antero-medial setae, three postero-medial setae, and two postero-apical setae; segment III armed with three claws, lengths 67.9 μm, 57.5 μm, and 52.1 μm, respectively.

***Mandible*** (Fig. 5C) with two protopodal segments, i.e., the coxa and the basis, the coxa nearly trapezoidal and the basis smaller and nearly rectangular. The exopodal branchial plate greatly reduced and unsegmented with two postero-medial setae and four apical setae. The endopodal part of the palp well-developed and comprising of three segments. Armature of endopod segments as follows: segment I with two postero-proximal setae, and one antero-distal seta and two postero-distal setae; segment II with three antero-medial setae and one postero-apical seta; segment III with three antero-apical setae.

***Maxillula*** (Fig. 5D) with a protopod. The protopod bearing three endites, each armed with four or five setae. The exopod forming a well-developed branchial plate. The endopod constituting a palp with two segments, of which segment I bearing three postero-apical setae and segment II ornamented with four apical setae.

***First thoracopod*** (Fig. 5E) uniramous with a protopod, length 64.3 μm (Table 3), and developing two anterior setae, one posterior seta and one or two apical seta(e). The endopod composed of four segments, of which proximal three segments being 51.6 μm, 24.7 μm, and 27.9 μm in length. Segment III ornamented with two different morphological characteristics, one with a claw which segment IV fused with the terminal claw ~ 60.0 μm in length, and the other bearing two setae, of which one approximately twice as long as the other, with lengths 81.4 μm and 40.4 μm, respectively (see the Suppl. material 1: Fig. S1).

***Second thoracopod*** (Fig. 5F) uniramous, bearing a protopod with length 58.0 μm (Table 3) and armed with two anterior setae and one posterior seta, and one apical seta. The endopod composed of four segments, of which proximal three segments being 54.7 μm, 20.4 μm, and 29.6 μm in length. Segment I bearing one antero-apical seta and segment IV fused with the terminal claw, being 67.7 μm in total length.

***Third thoracopod*** (Fig. 5G) uniramous with a protopod, with a length of 62.0 μm (Table 3), and with two anterior setae and one apical seta. The endopod composed of four segments, of which proximal three segments being 57.0 μm, 25.2 μm, and 25.7 μm in length. Segment I bearing one antero-apical seta and segment IV fused with the terminal claw, being 95.4 μm in length.

***Uropodal rami*** (Fig. 5H) reduced to a single segment, 39.2 μm in length (Table 3), bearing one postero-medial seta and one terminal seta.

## Discussion

### Life stages of *L. inopinata*

Like other arthropods, ostracods grow by moulting. From egg to adult, the podocopid Ostracoda usually undergo eight moulting stages. Many studies on life cycle have been published, including on *Heterocypris incongruens* Ramdohr, 1808 (Schreiber 1922), *Darwinula stevensoni* Brady & Robertson, 1870 (Scheerer-Ostermeyer 1940), *Cypridopsis vidua* O.F. Müller, 1776 (Kesling 1951), *Cyprideis torosa* Jones, 1850 (Weygoldt 1960), and *Eucypris virens* Jurine, 1820 (Smith and Martens 2000).

Based on our results, *L. inopinata* has a special growth pattern with a significant variation from Inner Mongolia populations at the transition from the instars A-5 to A-4. Carapace length is significantly positively correlated with the height (r = 0.994, P = 0.000). This phenomenon suggests that the shell length and height of *L. inopinata* are interdependent, which is in accordance with data on most other ostracod species, such as the study on *Eukloedenella adcapitisdolorella* (Forel 2015). However, in the ontogeny of *L. inopinata*, the instar A-5 is a very special growth stage, and there is no overlap in carapace size between the instars A-6, A-5, and A-4. This usually corresponds to significant development of soft parts, such as new appendages development (Meisch 2000; Smith and Martens 2000). Slight overlap occurs in other adjacent growth stages. Compared with the data published in Zhai et al. (2015), *L. inopinata* population living in MP has a less varied growth pattern, including the instars A-5 moulting to A-4. This indicates that there is an intraspecific difference in growth pattern of *L. inopinata*.

Yin et al. (1999) pointed out that *L. inopinata* populations, which is originally collected from Europe and China, has different morphological traits for both the valves (shape and size) and soft parts. Several studies suggested that the length of *L. inopinata* valves can be influenced by water salinity, i.e., species adapting to variable environment by adjusting body size for maintaining high fitness, a well-known life history strategy (Yin et al. 2001; Zhang et al. 2004; Xu et al. 2012). These studies only dealt with the specimens from Qinghai-Tibet Plateau. However, the difference in growth pattern between *L. inopinata* populations from Lake Jiang-Co and Lake Dali is possibly correlated with different water parameters, e.g., 0.59 vs. 7.60‰ salinity of Lake Jiang-Co and Lake Dali, respectively. It is reasonable to infer that the growth pattern of *L. inopinata* population living in a water with lower salinity condition is more volatile than in a higher salinity water body.

Brooks’ rule predicts that the growth ratio of crustaceans during its growth process is 1.26 (Brooks 1886; Teissier 1960). Although the mechanism of this pattern is still unclear, the growth of ostracods somewhat deviates from this prediction (e.g., Kesling and Takagi 1961; Kesling and Crafts 1962). Danielopol et al. (2008) reviewed these studies and recorded values ranging from 1.210 to 1.370 for non-marine ostracod species. However, an intraspecific difference is likely to exist in growth pattern of different populations. This may be due to different life history strategies, corresponding to variable environmental conditions. Additional studies on more species are necessary to prove this hypothesis.

### Comparisons between morphology of different *L. inopinata* populations

All the morphological characteristics of these populations have been compared with the European populations as described by Yin et al. (1999), Meisch (2000). Differences that occur in the chaetotaxy of the antenna, first thoracopod, and third thoracopod are significant.

Compared with the antenna of the European *L. inopinata* female (Yin et al. 1999; Meisch 2000), Lake Jiang-Co females have segment II carrying an additional postero-apical seta, while the chaetotaxy of the antenna is similar to the population studied by Yin et al. (1999). In our specimens, the protopod of the third thoracopod is unarmed, while there is one seta at the same place in the European *L. inopinata*.

The most obvious difference occurs in the first thoracopod. *Limnocythere inopinata* from Lake Jiang-Co has two types of chaetotaxy on the protopod and the terminal segment. One type is similar with the populations illustrated in Yin et al. (1999) and Meisch (2000), while the other is very different. The protopod of former one is armed with two apical setae and the terminal segment is fused with the claw it carries. In the latter type, the protopod bears only one apical seta and the terminal segment is armed with two setae. It is observed in one of the six dissected specimens. However, at the present moment with the lack of DNA data we cannot claim with great certainty that *L. inopinata* is actually a species complex.

## Conclusions

This study represents a bioculture experiment on *L. inopinata* in order to better understand its life stages and morphological variations from the A-8 to the adult. The living animals were collected from Lake Jiang-Co, northern Tibetan Plateau. *Limnocythere inopinata* has a special growth pattern in comparison to the prediction of Brooks’ rule and the growth pattern of *L. inopinata* population in Lake Dali, eastern MP. The maximum growth occurs in the instars A-5 moulting to the A-4. This indicates that difference in growth pattern between populations may be attributed to different life history strategies as adaptation to environmental conditions. The adults of *L. inopinata* from Lake Jiang-Co differ from European *L. inopinata* in the morphological characteristics of the appendages with the most obvious difference appearing in the first thoracopod.

## Supplementary materials

Supplementary material 1 Figure S1 Data type png image Explanation note Another morphological type of first thoracopod of *L. inopinata*, adult female. Protopod with one apical seta, and distal segment with two posterior setae, the well-developed seta approximately twice as long as smaller one. https://binary.pensoft.net/file/497774This dataset is made available under the Open Database License (http://opendatacommons.org/licenses/odbl/1.0/). The Open Database License (ODbL) is a license agreement intended to allow users to freely share, modify, and use this Dataset while maintaining this same freedom for others, provided that the original source and author(s) are credited.Can Wang, Hailei Wang, Xingxing Kuang, Ganlin Guo
