# Supplementary figures and images for: Life stages and morphological variations of Limnocythere inopinata (Crustacea, Ostracoda) from Lake Jiang-Co (northern Tibet): a bioculture experiment
Source: Zookeys. 2021 Jan 18;1011:25–40. doi: 10.3897/zookeys.1011.56065 (PMC7835202; doi:10.3897/zookeys.1011.56065)

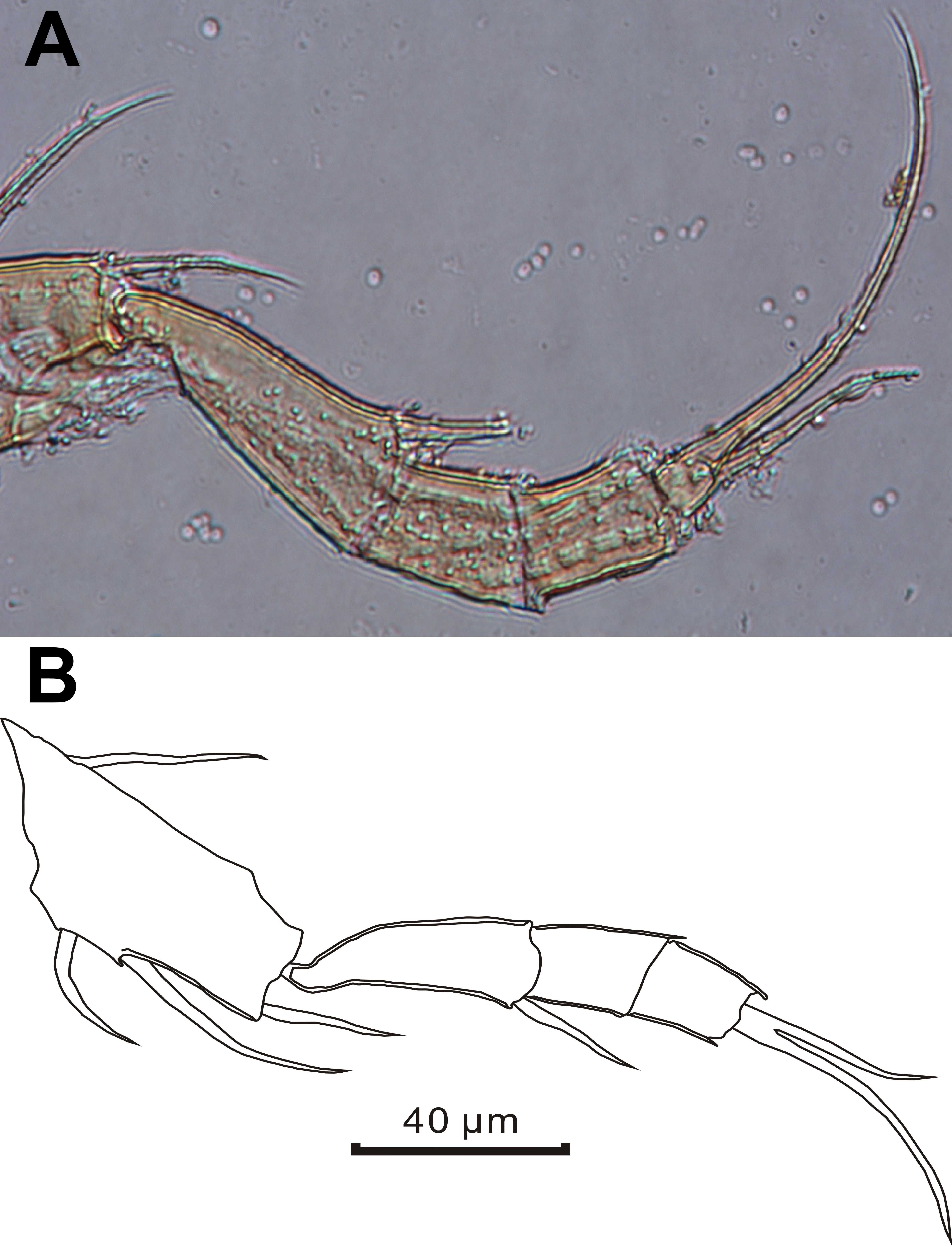

Supplement: Supplementary material 1 — Figure S1 [file zookeys-1011-025-s001.png]
